# GC-MS analysis of the methanolic extracts of *Smilax china* and *Salix alba* and their antioxidant activity

**DOI:** 10.3906/kim-1907-5

**Published:** 2020-04-01

**Authors:** Abdul QADIR, Mohd AQIL, Athar ALI, Farhan. J. AHMAD, Sayeed AHMAD, Muhammad ARIF, Nausheen KHAN

**Affiliations:** 1 Department of Pharmaceutics, School of Pharmaceutical Education & Research, Jamia Hamdard, New Delhi, India; 2 Centre for Transgenic Plant Development, Department of Biotechnology, Jamia Hamdard, New Delhi India; 3 Department of Pharmacognosy and Phytochemistry, School of Pharmaceutical Education & Research, Jamia Hamdard, New Delhi India; 4 Faculty of Pharmacy, Integral University, Lucknow India; 5 Transformative Learning Solution Private Limited, New Delhi India

**Keywords:** *Smilax china*, *Salix alba*, total antioxidant potential, DPPH assay, GC-MS

## Abstract

*Smilax china* L. (family Smilacaceae) and *Salix alba* L. (family Salicaceae) are plants that have been traditionally used to treat various ailments in Indian and Chinese medicine. A quantitative estimation of the methanolic extracts of these plants was performed by GC-MS analysis to obtain insight into its phytoconstituents responsible for therapeutic action. The antioxidant potential of the methanol extracts of *Smilax china* (MESC) and *Salix alba* (MESA) were assessed with DPPH by using a UV spectrophotometer at a wavelength of 517 nm. The prevailing compounds found in MESC were lactam sugars including 2,5-dimethyl-2,4-dihydroxy-3(2H)-furanon (1.40%), 1,5-anhydro-6-deoxyhexo-2,3-diulose (4.33%), and alpha-methyl-1-sorboside (1.80%); the two alkaloids found were 1,4-methane-4,4a,5,6,7,8,9,9a-octahydro-10,10-dimethyl cyclohepta[d] pyridazine (0.87%) and 1,3,7-trimethyl-2,6-dioxopurine(0.54%); terpenes included deltacadinene (0.39%), terpineol, (+)-cedrol (22.13%), 3-thujanol (0.77%), and 9,10-dehydro-cycloisolongifolene (0.34%); fatty acids included cis-vaccenic acid (4.98%) and telfairic acid (1.10%); esters included 1,2,3-propanetriol diacetate (7.56%), 7-hexadecenoic acid, methyl ester (1.77%), eicosanoic acid, and methyl ester (0.95%); and glycerol included 1,2,3-propanetriol (28.75%). The interesting compounds found in MESA were reducing sugars like D-allose (4.40%) and pyrogallol (10.48%), alkaloids like caffeine (63.49%), and esters like methyl octadecanoate (0.53%). Both fractions revealed considerable antioxidant activity. The reported existing phenolic compounds and terpenes are responsible for the antioxidant activity of the plant extracts.

## 1. Introduction

Herbal drugs have attracted mankind since ancient times. The WHO estimates that about 70% to 80% of the populations of some Asian and African countries rely on traditional medicine as the main source of primary health care [1]. The unique perceived benefits of these medicines include their effectiveness, easy availability, limited or no side effects, and success in the treatment of multiple chronic diseases. All these factors play a significant role in the widespread acceptance of natural or alternative therapies by the international community. To harness the benefits and employ intense applications in healthcare, they have been included in the pharmacopoeias of different countries for quality control purposes. In fact, many countries are making separate standard books for these herbs [2]. There is a sharp rise in the acceptance of herbal-based medicines due to lower toxicity issues and fewer or no side effects, which is a major issue in synthetic-based medicines. The amalgamation of modern science (delivery systems) with herbal drugs ensures standardization, consistent and safer use, and regulation of quality control [3]. Unlike synthetic medicines, where combinations are usually avoided, herbal medicines have found popularity as polyherbal formulations. Literature survey reports reveal that these polyherbal preparations have multiple active constituents, which act in a synergistic manner to give a combined effect within the human body [4]. *Smilax china* L. (family Smilacaceae) and *Salix alba* L. (family Salicaceae) are two popular herbal drugs used in various polyherbal preparations in the Indian and Chinese systems of medicine. They have been incorporated for their abundant therapeutic effects. *S. china* is a deciduous tree with rounded leaves and red berries [5]. Quercetin is reported to be the bioactive component, which is also a marker compound of this plant [6]. *S. alba* is ordinarily known as willow bark or white willow [7]. Salicin is the major component of this plant. It is a metabolic precursor of salicylic acid, which possesses activity similar to aspirin. It is reported to possess antipyretic and analgesic effects [8]. Gas chromatography coupled with mass spectrometry (GC-MS) makes it a high-throughput dual analytical tool. This combination leads to high chromatographic tenacity and peak intensity and provides compositional information for nearly all volatile and semivolatile materials, including organic acids, amino acids, and so on. In this context, the bioactive components present in *Smilax china* and *Salix alba* were identified by GC-MS analysis in the present study [9]. The vital components in these plants can be characterized and explored as valuable assets in the pharmaceutical and nutraceutical industries. In the present study, extraction of these plant materials was carried out followed by screening of the antioxidant potential of the extracts.

## 2. Materials and methods

### 2.1. Collection and authentication of seeds

Chob chini rhizome ( *S. china* ) and white willow bark ( *S. alba* ) were procured from a local market of Delhi, India, in March 2018 and were identified by R.S. Jayasomu, Head, Dept. of Herbarium and Museum, National Institute of Science Communication and Information Resources (NISCAIR), New Delhi. The authentication numbers assigned were NISCAIR/RHMD/Consult/-2019/3402-03-2 ( *S. china* ) and NISCAIR/RHMD/Consult/2019/3402-03-1 ( *S. alba* ).

### 2.2. Chemicals

1,1-Diphenyl-2-picrylhydrazyl (DPPH) was bought from Sigma-Aldrich Pvt. Ltd. (Mumbai) and the solvents used for extraction, fractionation, and purification were of analytical grade and procured from Merck (Mumbai).

### 2.3. Extraction of plant material

Plant material (200 g) was defatted with 500 mL of n-hexane for 24 h with occasional shaking. Afterwards, the drug was filtered, dried, and loaded in a Soxhlet apparatus for the extraction process. The drug was extracted in segments; as one batch, 40 g of crude drug was loaded in the Soxhlet apparatus with 120 mL of methanol at 55–60 °C and continuously extracted for 6 h. Similarly, another batch of crude drug was extracted. Following complete extraction, the solvent was evaporated under reduced pressure to obtain a solid mass (extract) and then lyophilized to obtain the solid powder form (18.7 g MESC and 9.9 g MESA). A reported method was used to analyze minor quantities (1 g) of both extracts via GC-MS [10].

### 2.4. Phytochemical screening

Initial phytochemical investigation of the methanolic extracts of *Smilax china* (MESC) and *Salix alba* (MESA) was done for the presence of various phytoconstituents like alkaloids, phenolic, flavonoids, steroids, reducing sugar, saponins, carbohydrate, gums, glycosides, and tannins [11].

### 2.5. GC-MS analysis of bioactive compounds

MESC and MESA were analyzed by GC-MS. The GC-MS device consisted of a Shimadzu QP-2010 Ultra with a capillary standard and nonpolar column 60 M TRX 5-MS (dimension: 30 m, ID: 0.25 mm, film: 0.25 mm). The carrier gas was helium with mobile phase flow rate set at 1.21 mL min^-1^ . The temperature of the instrument’s oven was raised from 100 °C to 260 °C at a rate of 10 °C min^-1^ and the volume per injection was set at 2 μL. In GC-MS, an electron ionization energy system was used with 70 eV. The total running time was 65 min for both samples, *Smilax china* and *Salix alba* . The MESC and MESA were dissolved in methanol and run completely in a range of 10–850 m/z. The results were analyzed and equated using the Wiley spectral library search program. Within a time span of 30–35 min, the mass spectra were obtained [10]. Various aspects of compound identification like molecular formula, molecular weight, and structure of the bioactive components of test materials along with their names were established while the comparative percentage of each component was calculated by equating its average peak area to the total areas. The identification of the individual compounds was carried out by comparing the m/z ratios with those samples authenticated by Sigma-Aldrich along with mass spectra data in the NIST Mass Spectral Library Ver. 2.0 d (2005) and the literature [12].

### 2.6. Antioxidant activity

The radical scavenging potential of MESC and MESA against 1,1-diphenyl-2-picrylhydrazyl (DPPH) were determined using an analytical technique and UV spectrophotometer at 517 nm. Aliquots (40, 60, 80, 100, 120, and 140 μg/mL for MESC and MESA) were dissolved in test tubes containing 3 mL of methanol and 0.5 mL of 1 mM DPPH. Vitamin E (α-tocopherol) was used as the standard with the same concentrations as the test samples. A placebo solution with equal volume of methanol and DPPH was made and this solution mixture was incubated at room temperature for 30 min [13]. The antioxidant activity was quantified using the following equation:

% Scavenging activity = ((Ab – As)/Ab) ×100,

where Ab denotes the absorbance of the blank and As denotes the absorbance of the sample.

All experimentation was done in triplicate. The values of the calculated IC_50_ are expressed as mean ±SD.

## 3. Results and discussion

### 3.1. Analysis of bioactive compounds

**Figure 1:**
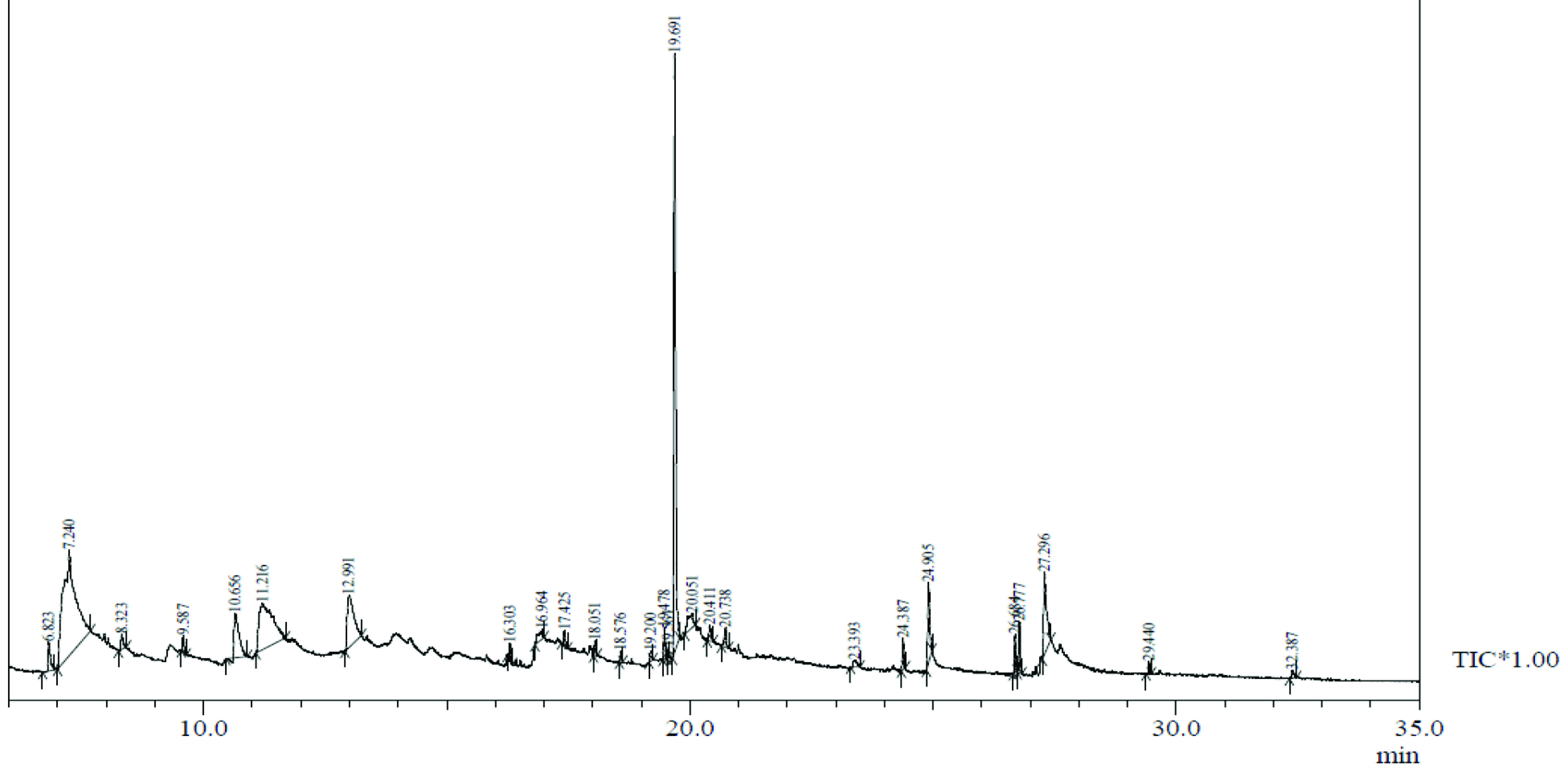
GC-MS chromatogram of the methanolic extract of *Smilax china*

**Figure 2:**
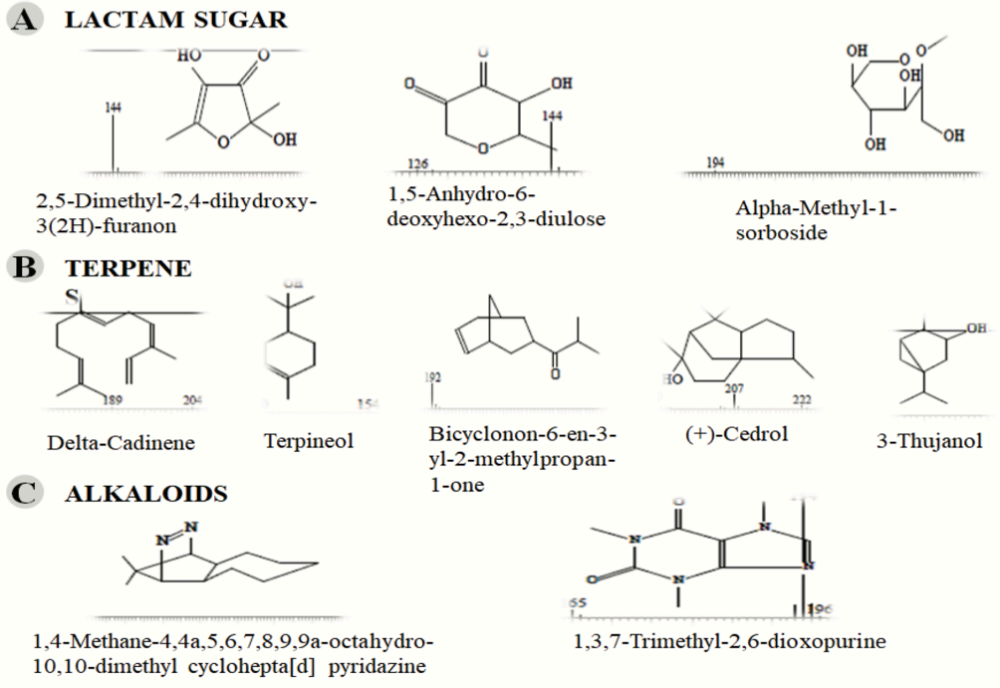
Chemical compound hits on GC-MS chromatogram of methanolic extract of *Smilax china* .

**Figure 3:**
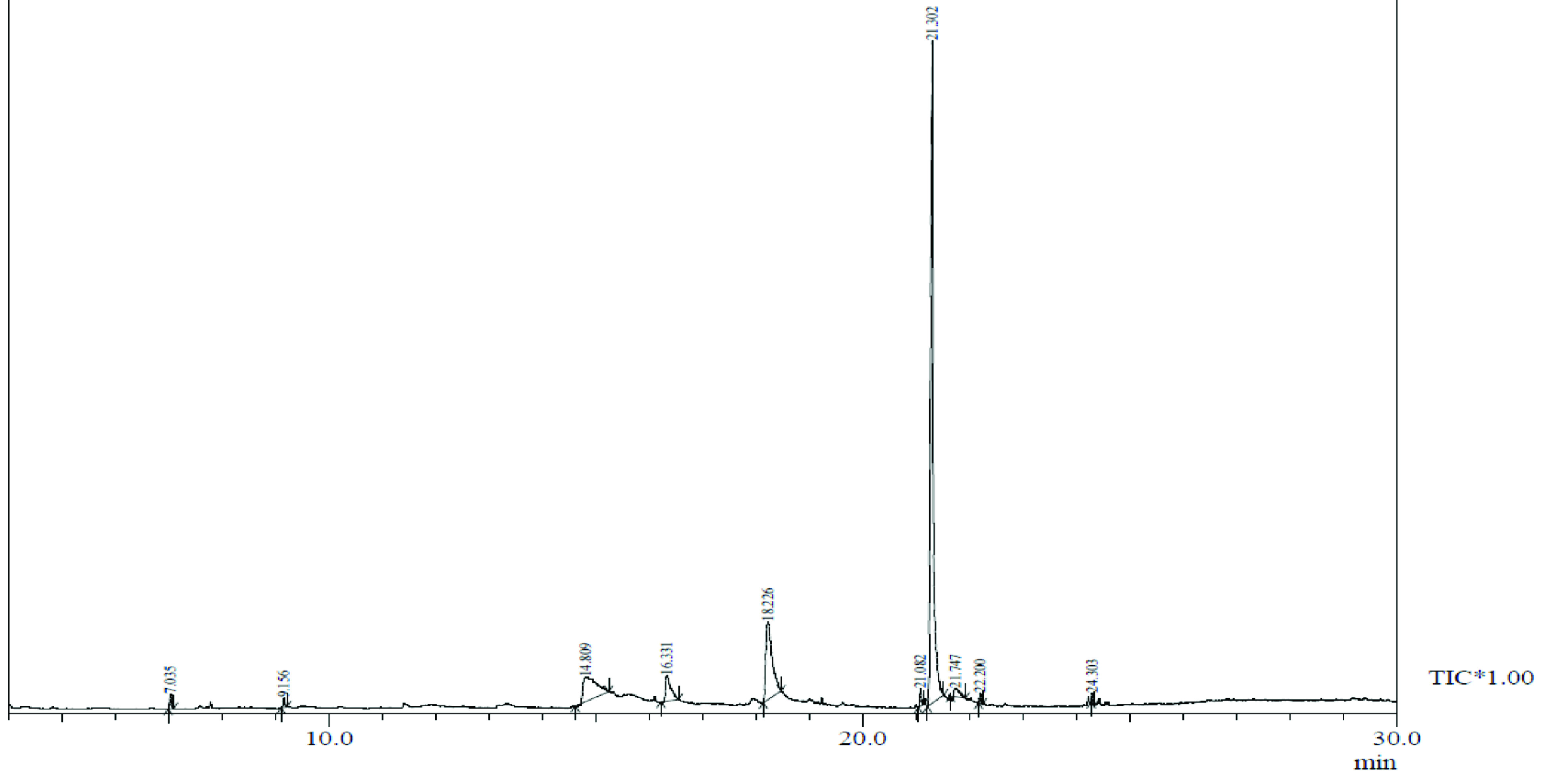
GC-MS chromatogram of methanolic extract of *Salix alba*

**Figure 4:**
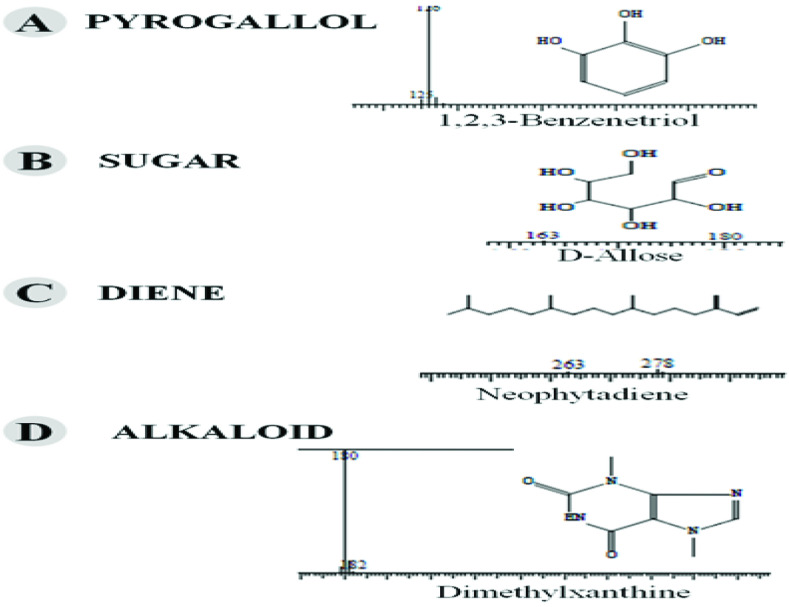
Chemical compound hits on GC-MS chromatogram of methanolic extract of *Salix alba* .

The extraction and analysis of plant material plays a vital role in the development of herbal formulations, which includes quality control and modernization of herbal drugs. Henceforth, the present study was designed to obtain the bioactive compounds present in MESC and MESA using GC-MS [14]. Crude drug powders of *Smilax china* and *Salix alba* were defatted with n-hexane before extraction with methanol for easy extraction and identification of important bioactive components. Phytochemical tests for the methanolic extracts of both plants showed the presence of reducing sugar, steroids, flavonoids, alkaloids, terpenes, and fatty esters. In *S. china* and *S. alba* , a total of 26 and 10 types of bioactive compounds have been identified, respectively. The GC-MS chromatograms obtained for MESC and MESA are shown in Figure 1 and 2, respectively. The active components, peak area, concentration (%), and retention time are exhibited in Table 1 and Table 2. The prevailing compounds found in the methanolic extract of *Smilax china* were lactam sugars like 2,5-dimethyl-2,4-dihydroxy-3(2H)-furanon (1.40%), 1,5-anhydro-6-deoxyhexo-2,3-diulose (4.33%), and alpha-methyl-1-sorboside (1.80%); alkaloids like 1,4-methane-4,4a,5,6,7,8,9,9a-octahydro-10,10-dimethyl cyclohepta[d] pyridazine (0.87%) and 1,3,7-trimethyl-2,6-dioxopurine(0.54%); terpenes like delta-cadinene (0.39%), terpineol, (+)-cedrol (22.13%), 3-thujanol (0.77%), and 9,10-dehydro-cycloisolongifolene (0.34%); fatty acids like cis-vaccenic acid (4.98%) and telfairic acid (1.10%); esters like 1,2,3-propanetriol di-acetate (7.56%), 7-hexadecenoic acid, methyl ester (1.77%), eicosanoic acid, and methyl ester (0.95%); and glycerol like 1,2,3-propanetriol (28.75%) (Figure 3). The compounds found in the methanolic extract of *Salix alba* were reducing sugars like D-allose (4.40%) and pyrogallol (10.48%), alkaloids like caffeine (63.49%), and esters like methyl octadecanoate (0.53%) (Figure 4). Both MESC and MESA were found to contain different types of important chemical constituents, which were fatty acid esters, terpenes, high-molecular-weight hydrocarbons, and their oxygenated products. Fatty acids like cis-vaccenic acid and telfairic acid and esters are mainly observed in both plant extracts. Cis-vaccenic acid is a kind of trans-fatty acid (omega-7 fatty acid) found in human milk, known for its various biological effects like antibacterial and hypolipidemic effects in rats [15]. Linoleic acid is also known as telfairic acid, which is a polyunsaturated omega-6 fatty acid. It has been found to have antibacterial activity, particularly in inhibiting the growth of gram-positive bacterial species [16]. Pyridazine alkaloids like 1,4-methane-4,4a,5,6,7,8,9,9a-octahydro-10,10-dimethyl cyclohepta[d] pyridazine were identified in *Smilax china* and xanthine alkaloids like 1,3,7-trimethyl-2,6-dioxopurine were present in both plant extracts. These alkaloid compounds have various important medicinal activities like antimicrobial, antioxidant, and antiinflammatory properties and treatment of hypotension [17]. Terpenes like delta-cadinene, terpineol, (+)-cedrol, and 3-thujanol were present in *Smilax china* and these terpenoids have various important pharmacological activities like antitumor, antimicrobial, antiinflammatory, anthelminthic, antioxidant, antiepileptic, pesticidal, antigonorrheal, and antihyperglycemic activity [18,19]. Pyrogallol is a naturally occurring potent antipsoriatic and antioxidant drug with beneficial effects in the treatment of various skin disorders [20].

**Table 1:** Biologically active chemical compounds present in *Smilax china* using GC-MS analysis.

S. no.	RT	Peak area%	Name of compound	MF	MW	CAS No.	Compound's nature	Structure of compound
1	6.823	1.40	2,4-Dihydroxy-2,5-dimethyl-2,4-dihydroxy-3(2H)-furanon	C_6_H_8_O_4_	144	10230-62-3	Lactam sugar	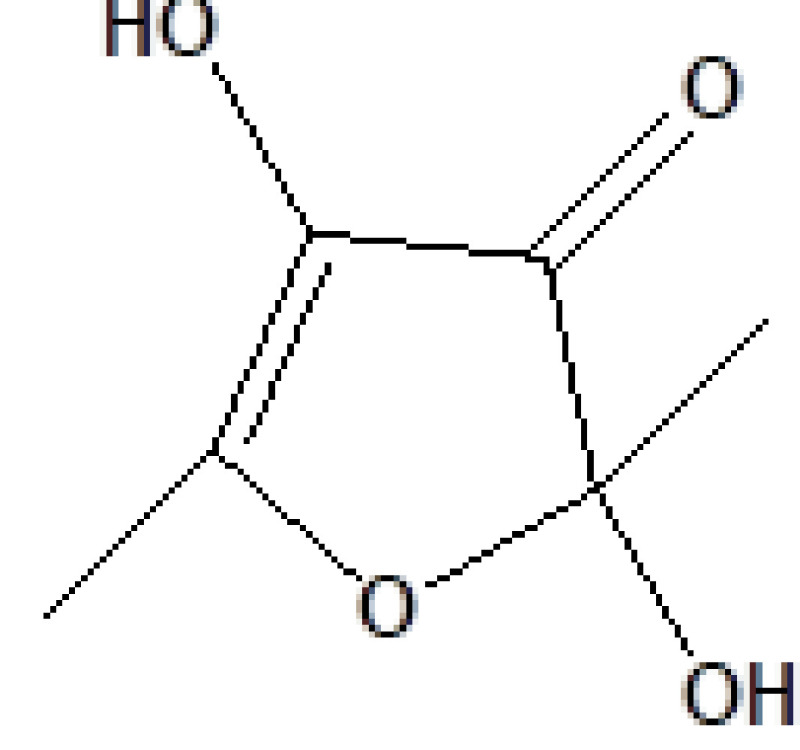
2	7.240	28.75	1,2,3-Propanetriol	C_3_H_8_O_3_	92	56-81-5	Glycerol	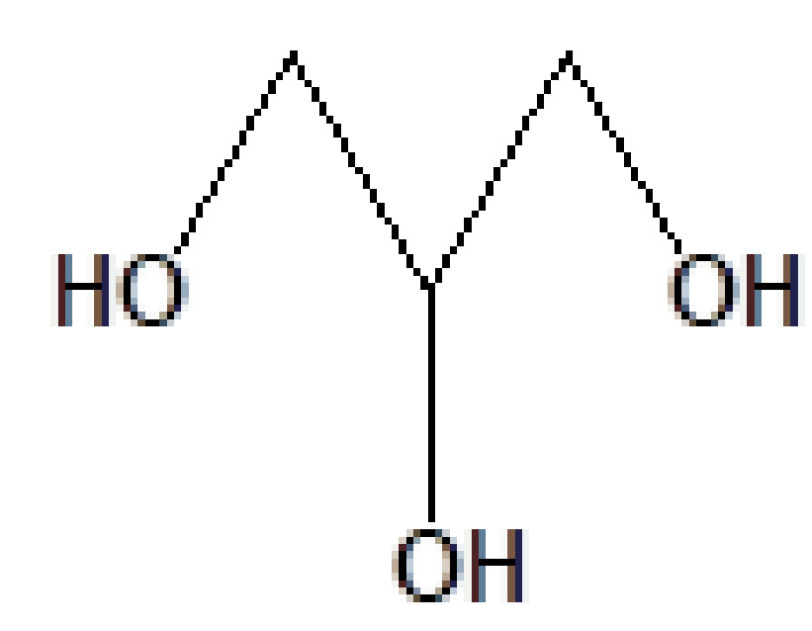
3	8.323	0.68	Acetic acid, butyl ester	C_6_H_12_O_2_	116	123-86-4	Ester	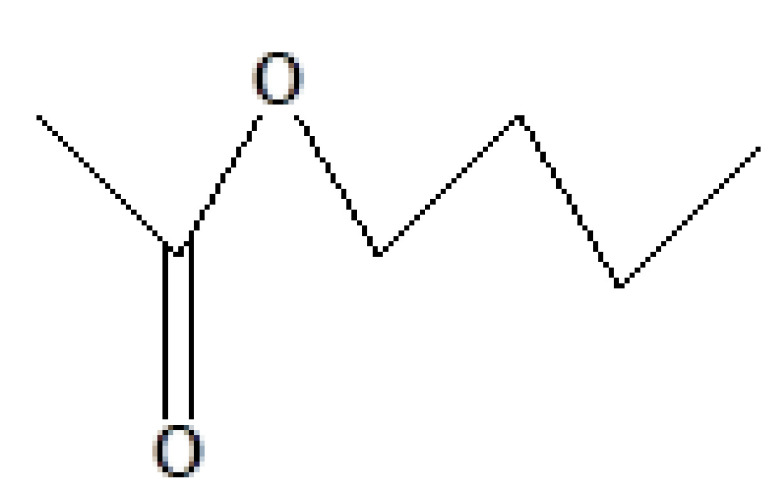
4	9.587	0.62	2,2-Dimethylbutane	C_6_H_14_	86	75-83-2	Hydrocarbon	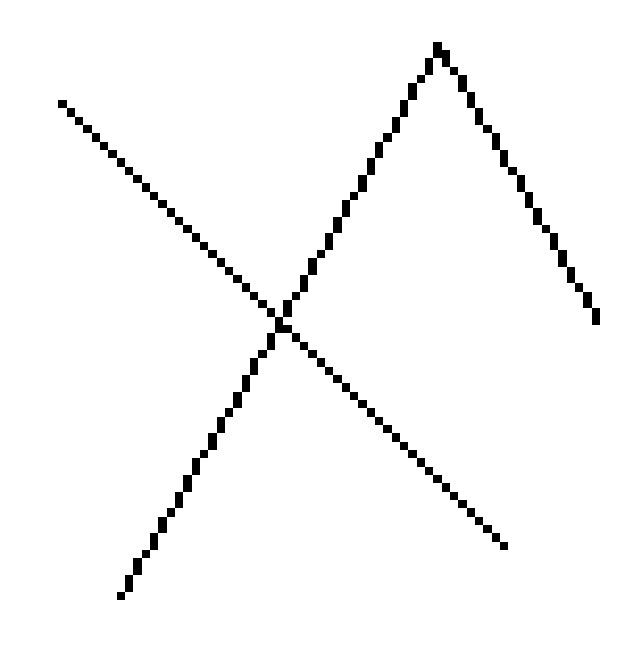
5	10.656	4.33	1,5-Anhydro-6-deoxyhexo-2,3-diulose	C_6_H_8_O_4_	144	28564-83-2	Sugar	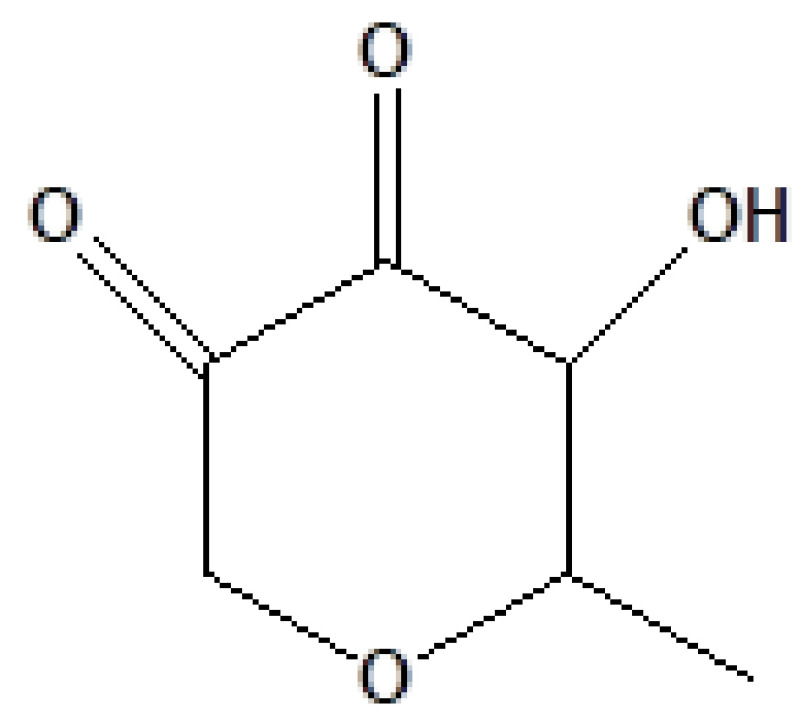
6	11.216	13.52	1,2,3-Propanetriol	C_3_H_8_O_3_	92	56-81-5	Glycerol	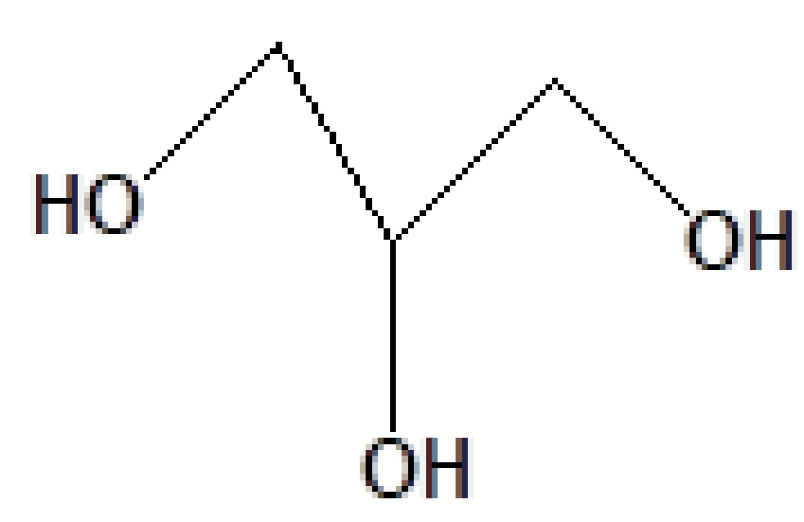
7	12.991	7.56	1,2,3-Propanetriol diacetate	C_7_H_12_O_5_	176	25395-31-7	Ester	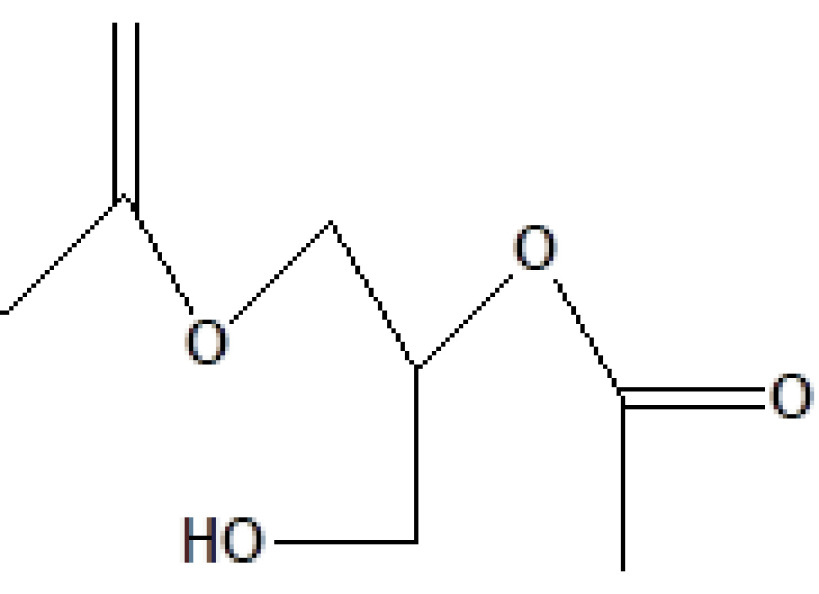
8	16.963	1.01	1,6-Octadien-3-ol, 3,7-dimethyl-isovalerate	C_15_H_26_O_2_	238	1118-27-0	Terpenes	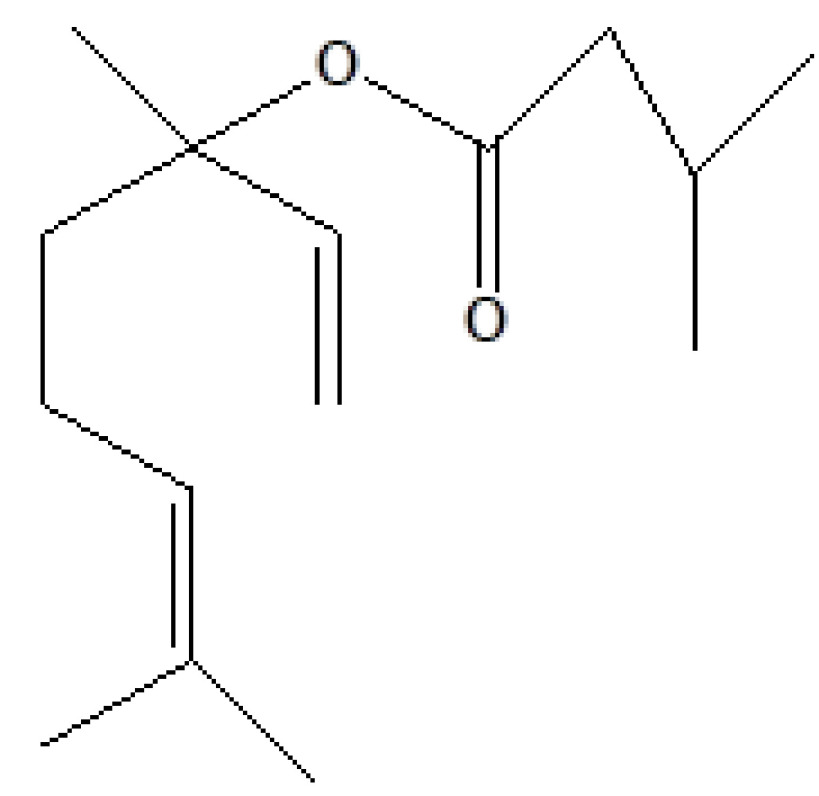
9	17.425	0.47	1-Chlorotetradecane	C_14_H_29_Cl	232	2425-54-9	Hydrocarbon	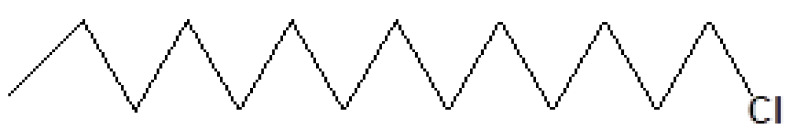
10	18.051	0.39	Delta-cadinene	C_15_H_24_	204	483-76-1	Terpenes	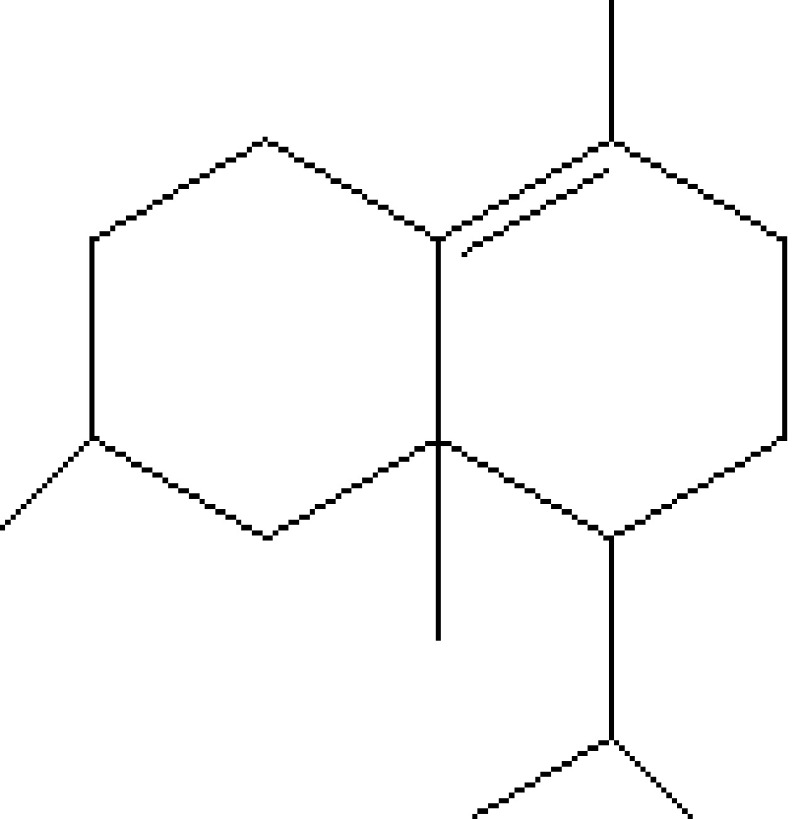
11	18.576	0.32	Terpineol	C_10_H_18_O	154	10482-56-1	Terpenes	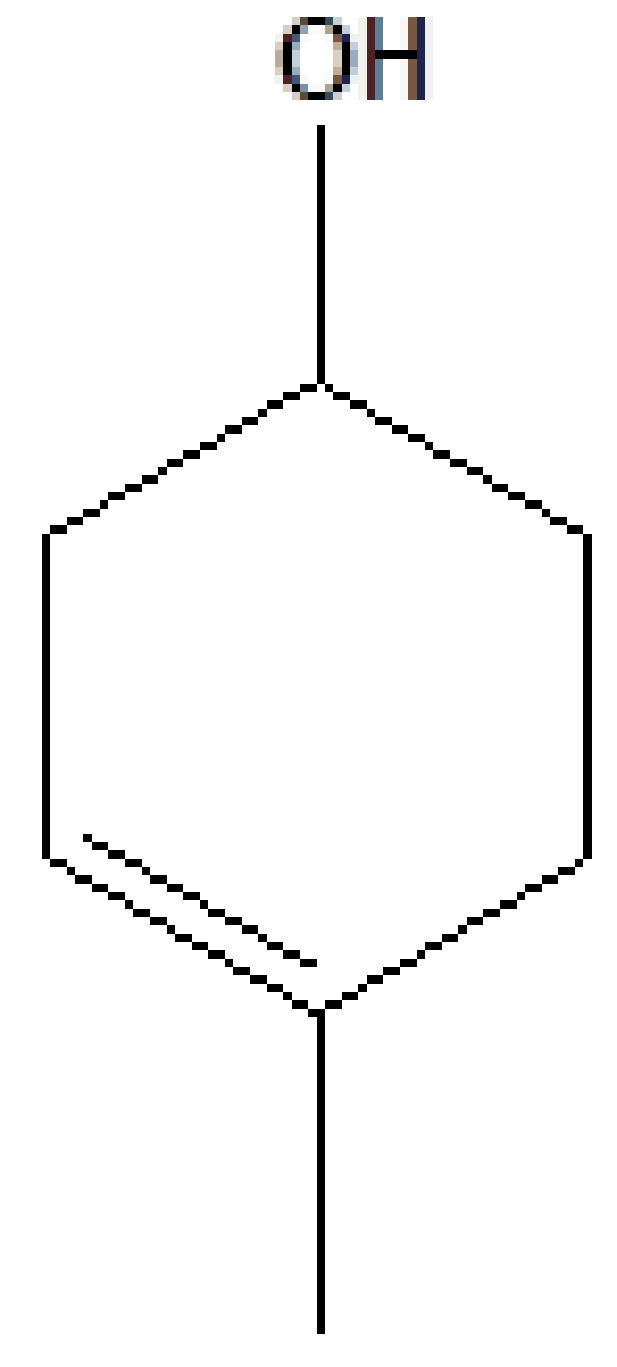
12	19.200	0.32	Bicyclonon-6-en-3-yl-2-methylpropan-1-one	C_13_H_20_O	192	103258-73-7	Terpenes	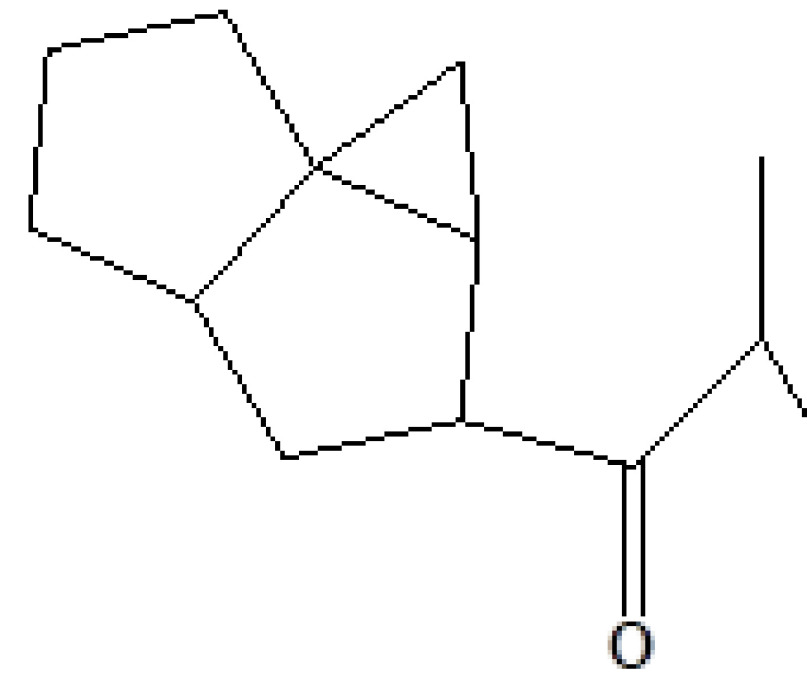
13	19.478	0.87	1,4-Methane-4,4a,5,6,7,8,9,9a-octahydro-10,10-dimethyl cyclohepta[d] pyridazine	H_12_H_20_N_2_	192	0-00-0	Alkaloid	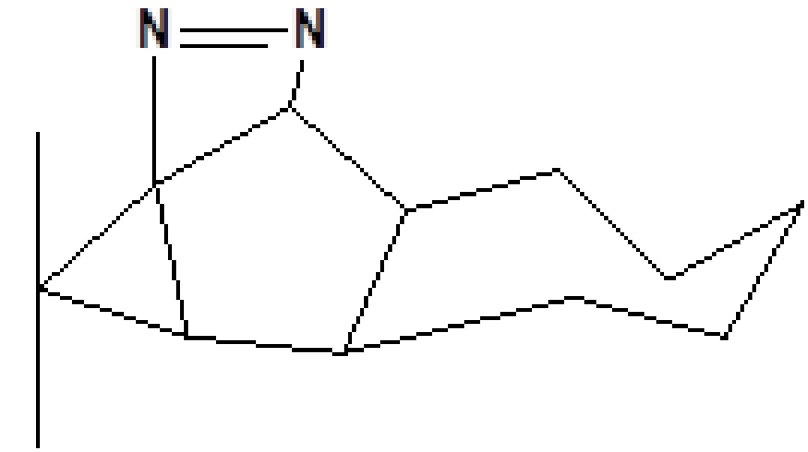
14	19.561	0.21	8-Isopropyl-1-methyl-5-methylene-1,6-cyclodecadiene	C_15_H_24_	204	23986-74-5	Terpenes	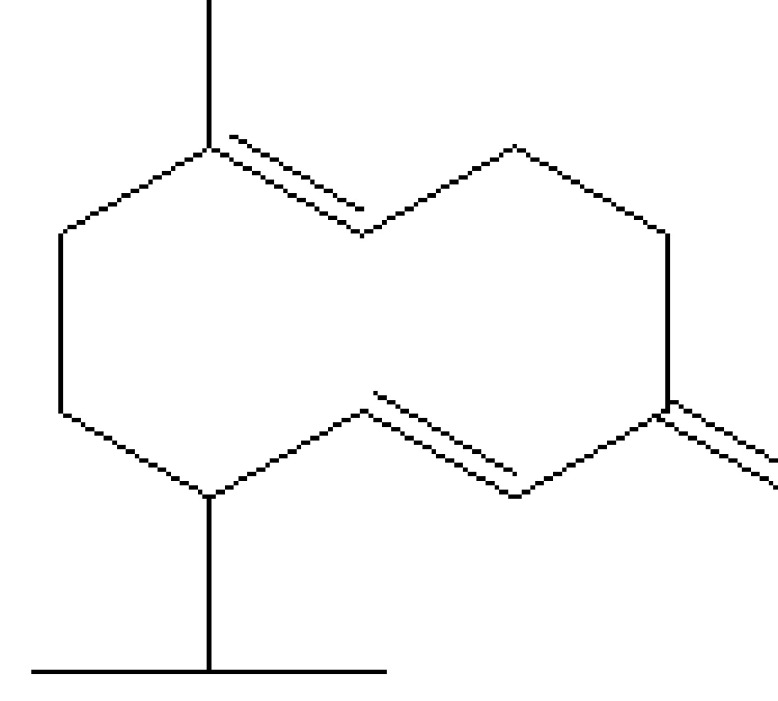
15	19.691	22.13	(+)-Cedrol	C_15_H_26_O	222	77-53-2	Terpenes	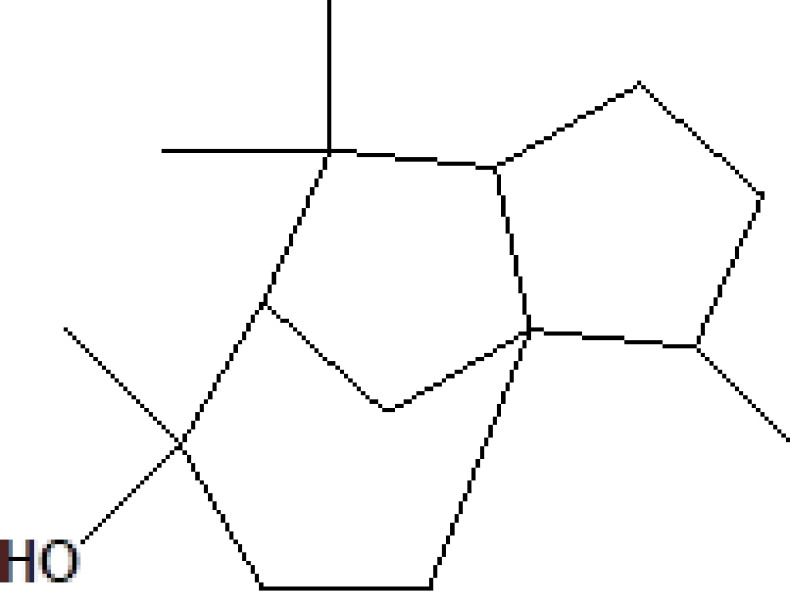
16	20.051	1.80	Alpha-methyl-1-sorboside	C_7_H_14_O_6_	194	3765-95-5	Sugar	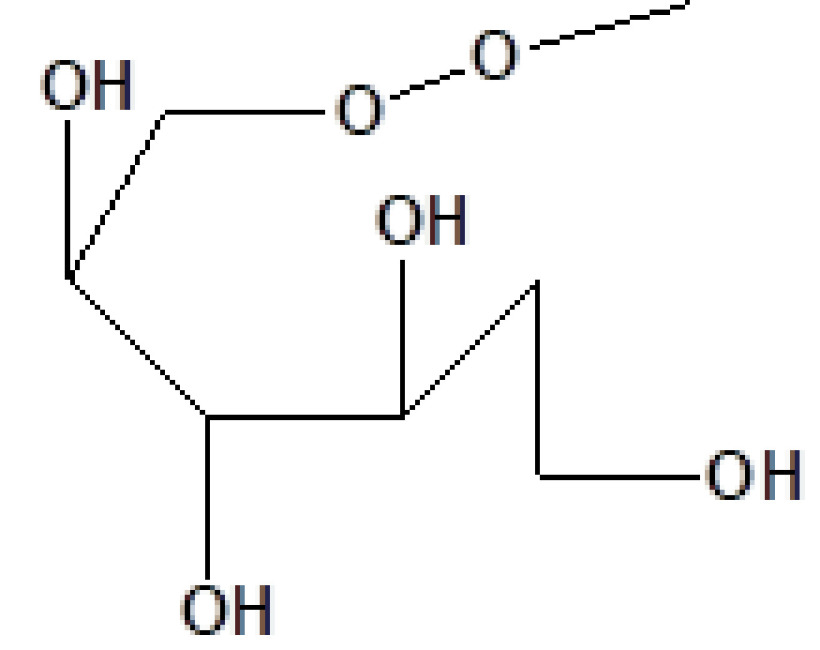
17	20.411	0.77	3-Thujanol	C_10_H_18_O	154	513-23-5	Terpenes	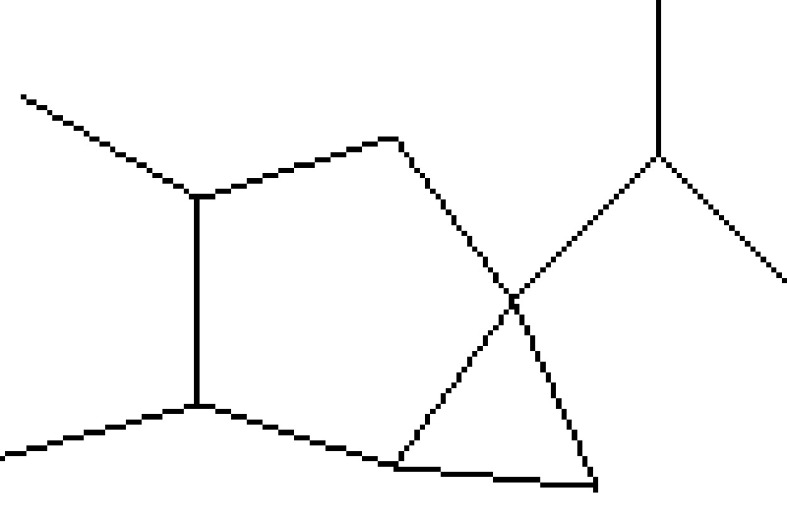
18	20.738	0.87	1-Octadecyne	C_18_H_34_	250	629-89-0	Hydrocarbon	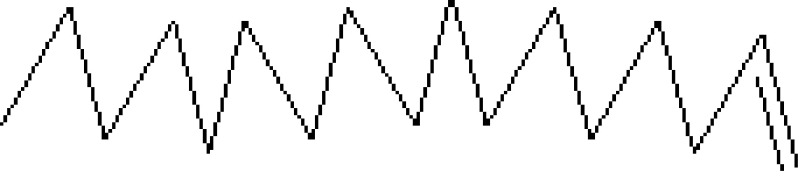
19	23.393	0.54	1,3,7-Trimethyl-2,6-dioxopurine	C_8_H_10_N_4_O_2_	194	58-08-2	Alkaloids	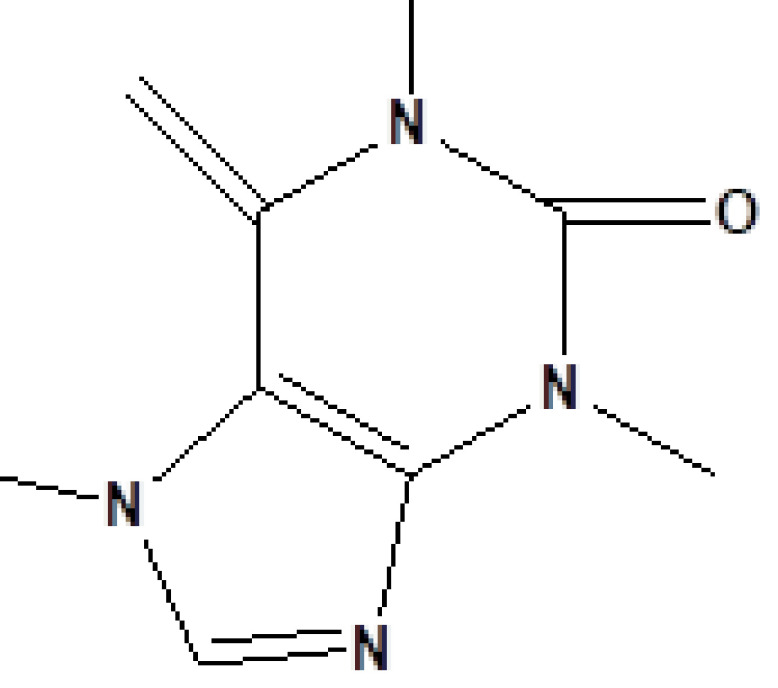
20	24.387	0.95	Eicosanoic acid, methyl ester	C_21_H_42_O_2_	326	1120-28-1	Fatty acid ester	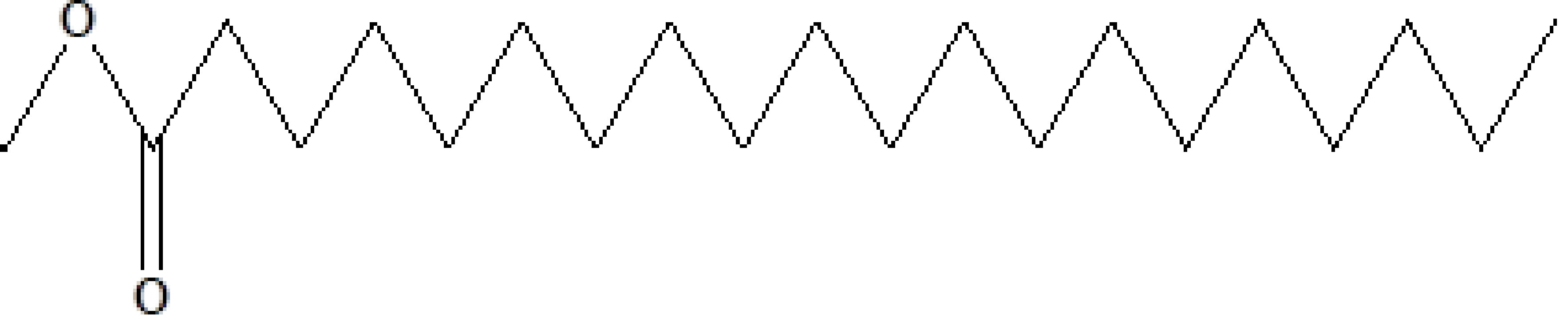
21	24.905	3.58	n-Hexadecanoic acid	C_16_H_32_O_2_	256	57-10-3	Fatty acids	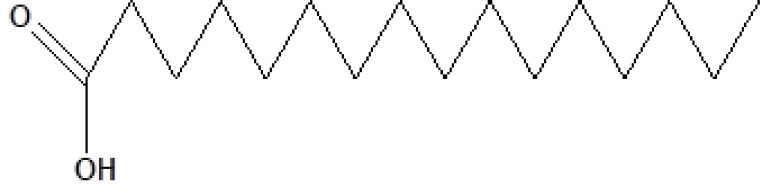
22	26.684	1.10	Telfairic acid	C_18_H_32_O_2_	280	60-33-3	Fatty acids	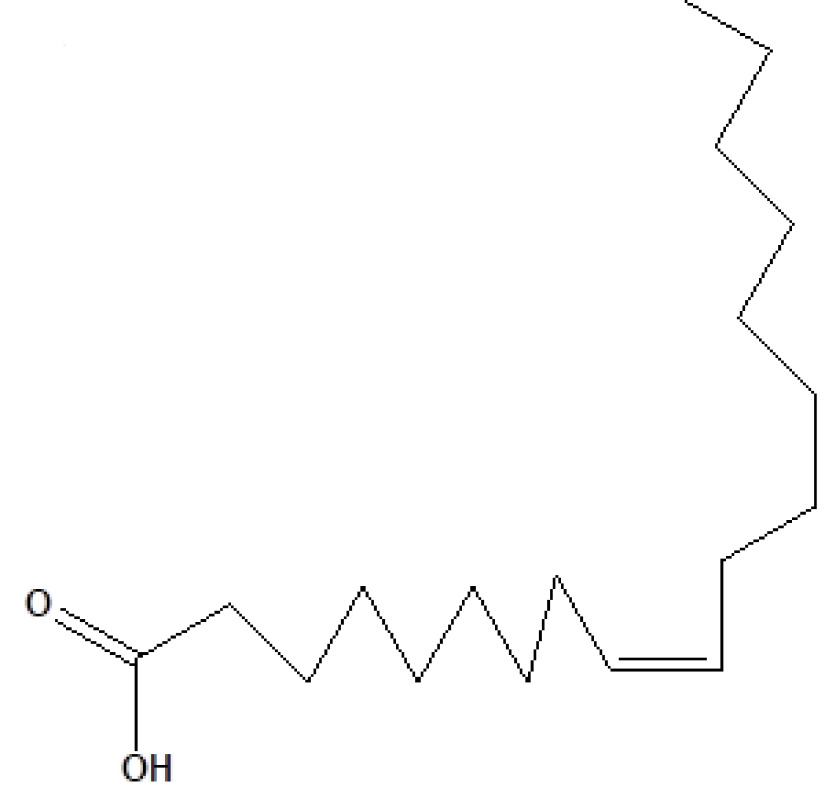
23	26.777	1.77	7-Hexadecenoic acid, methyl ester	C_17_H_32_O_2_	268	56875-67-3	Fatty acid ester	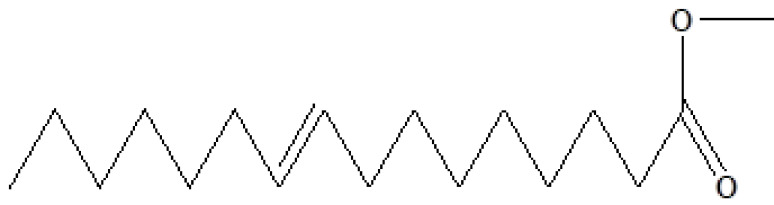
24	27.296	4.98	Cis-vaccenic acid	C_18_H_34_O_2_	282	506-17-2	Fatty acids	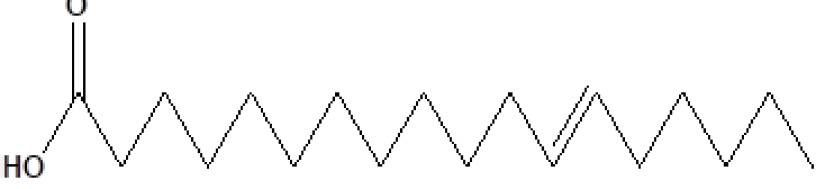
25	29.440	0.34	9,10-Dehydro-cycloisolongifolene	C_15_H_22_	202	0-00-0	Terpenes	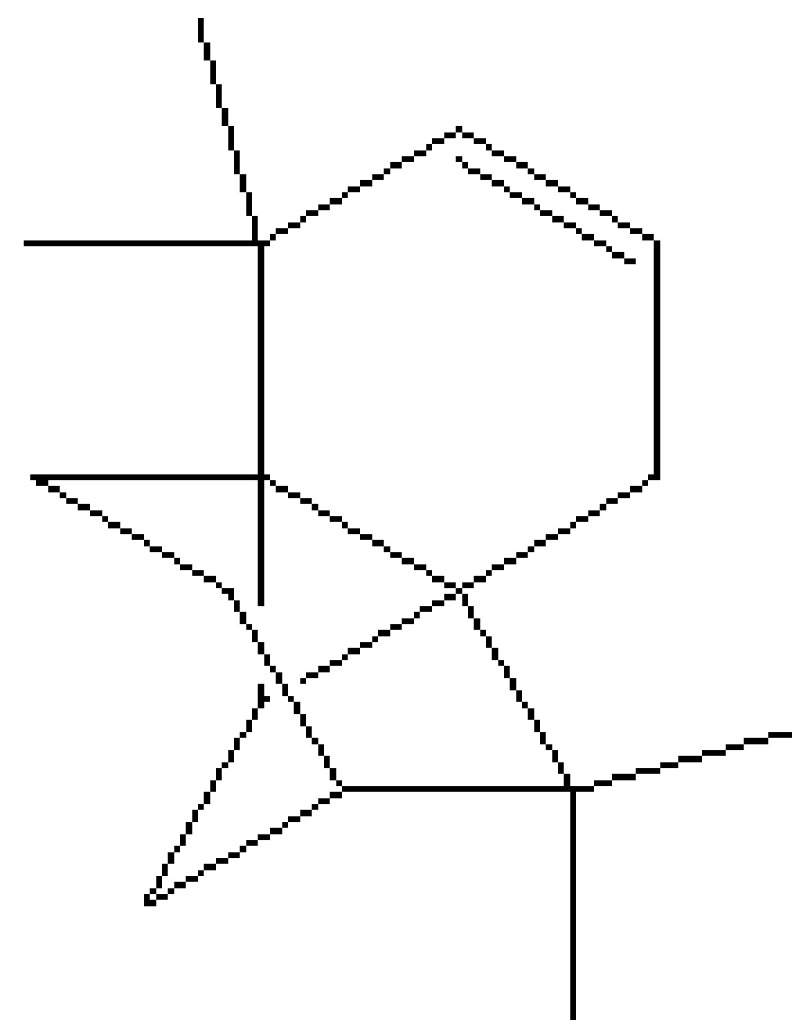
26	32.387	0.38	1,2-Benzenedicarboxylic acid, dioctyl ester	C_24_H_38_O_4_	390	117-84-0	Prostaglandin	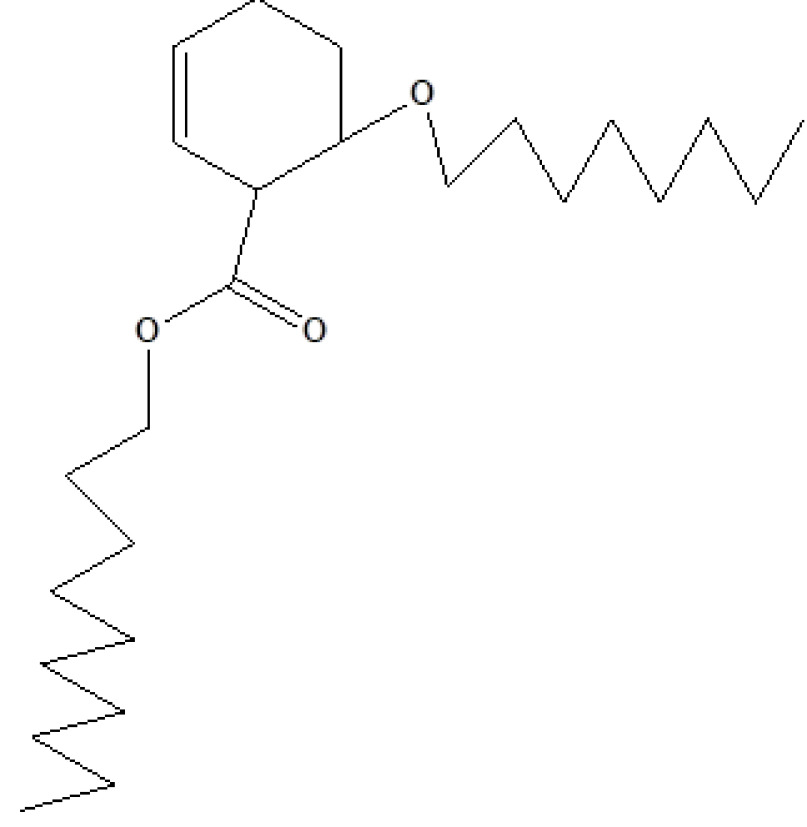

**Table 2:** Biologically active chemical compounds present in *Salix alba* using GC-MS analysis.

S. no.	RT	Peak area%	Name of compounds	MF	MW	CAS no.	Compound.s nature	Structure of compound
1	7.035	1.01	Undecane	C_11_H_24_	156	1120-21-4	Hydrocarbon	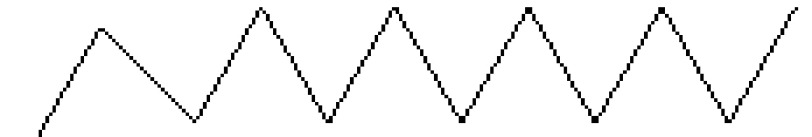
2	9.156	0.53	Tridecane	C_13_H_28_	184	629-50-5	Hydrocarbon	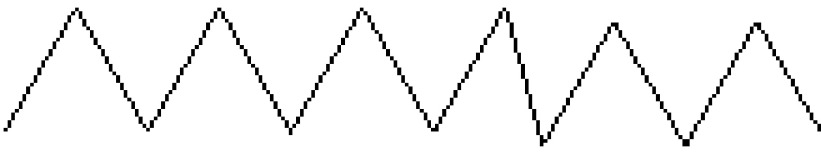
3	14.809	10.48	1,2,3-Benzenetriol	C_6_H_6_O_3_	126	87-66-1	Pyrogallol	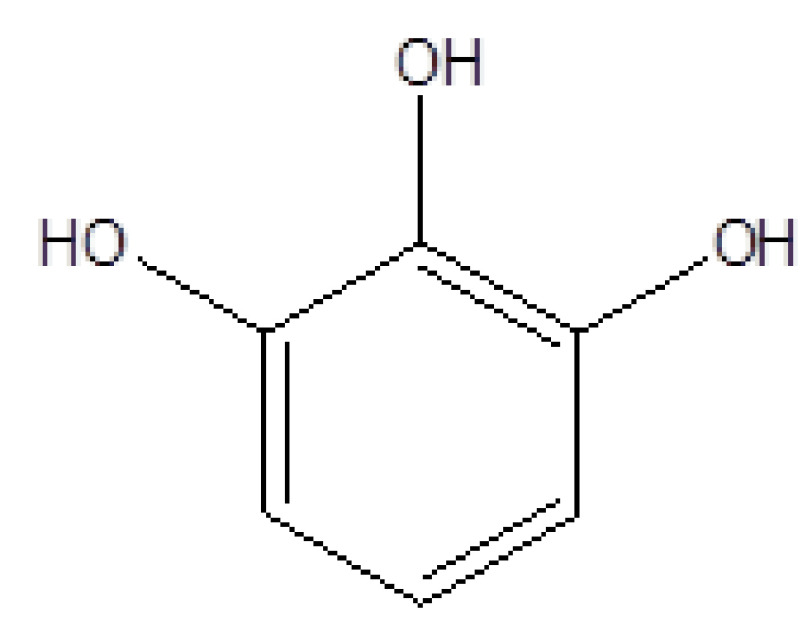
4	16.331	4.40	D-Allose	C_6_H_12_O_6_	180	2595-97-3	Sugar	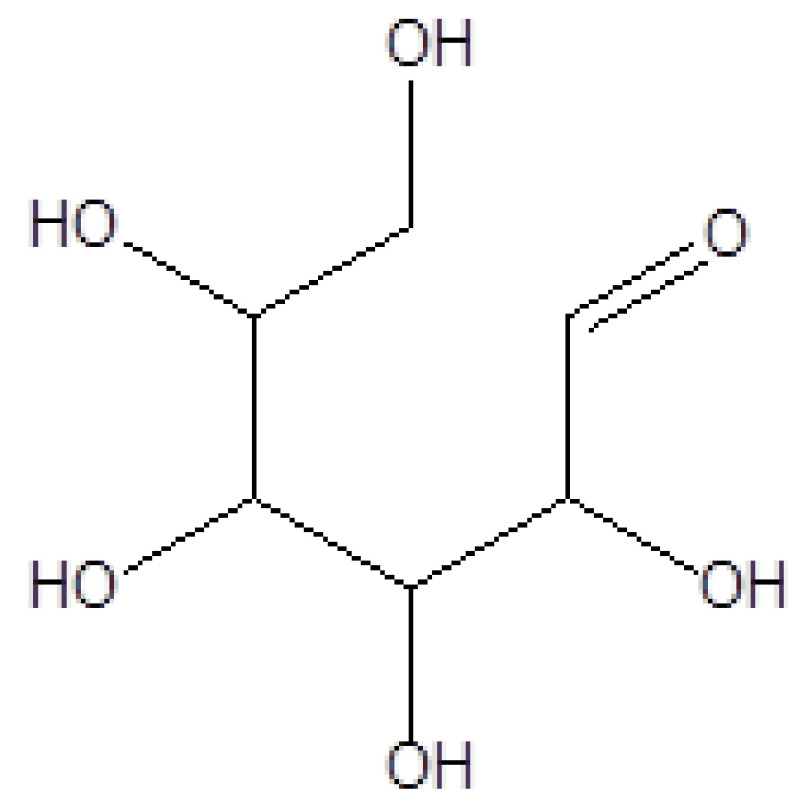
5	18.226	16.77	1,3,4,5-Tetrahydroxy-cyclohexanecarboxylic acid	C_7_H_12_O_6_	192	77-95-2	Alicyclic compound	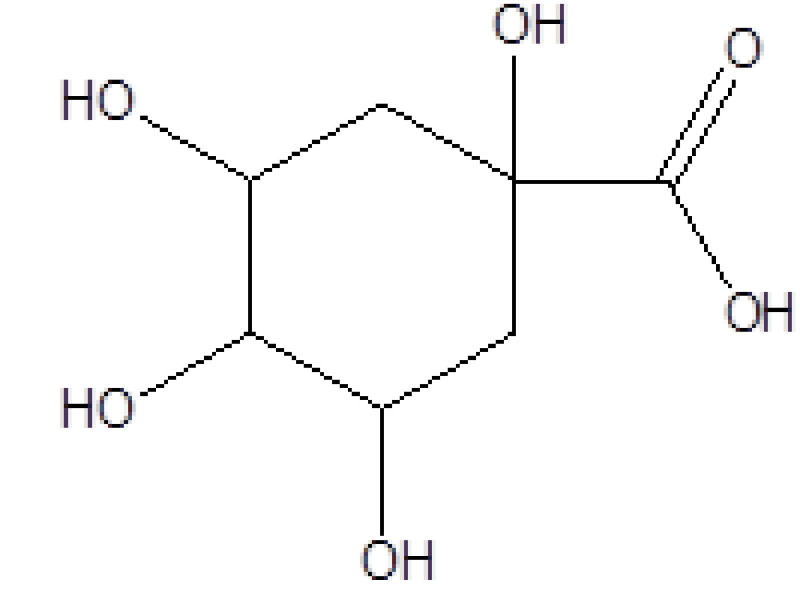
6	21.082	0.93	Neophytadiene	C_20_H_38_	278	504-96-1	Diene	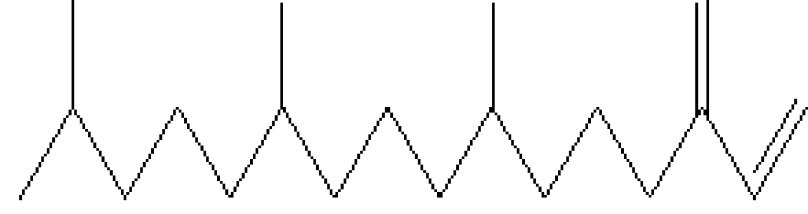
7	21.302	63.49	Caffeine	C_8_H_10_N_4_O_2_	194	58-08-2	Alkaloid	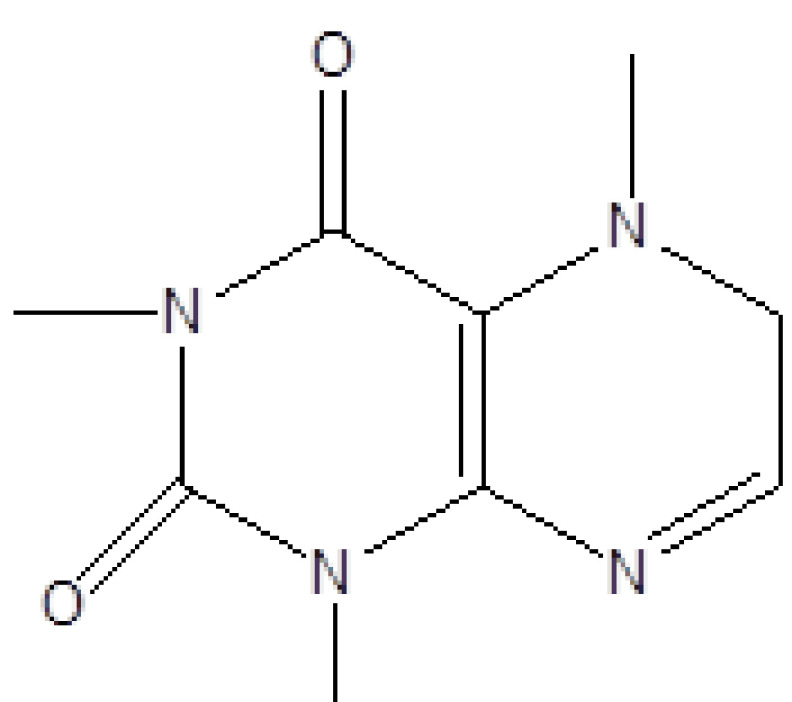
8	21.747	1.38	Dimethylxanthine	C_7_H_8_N_4_O_2_	180	83-67-0	Alkaloid	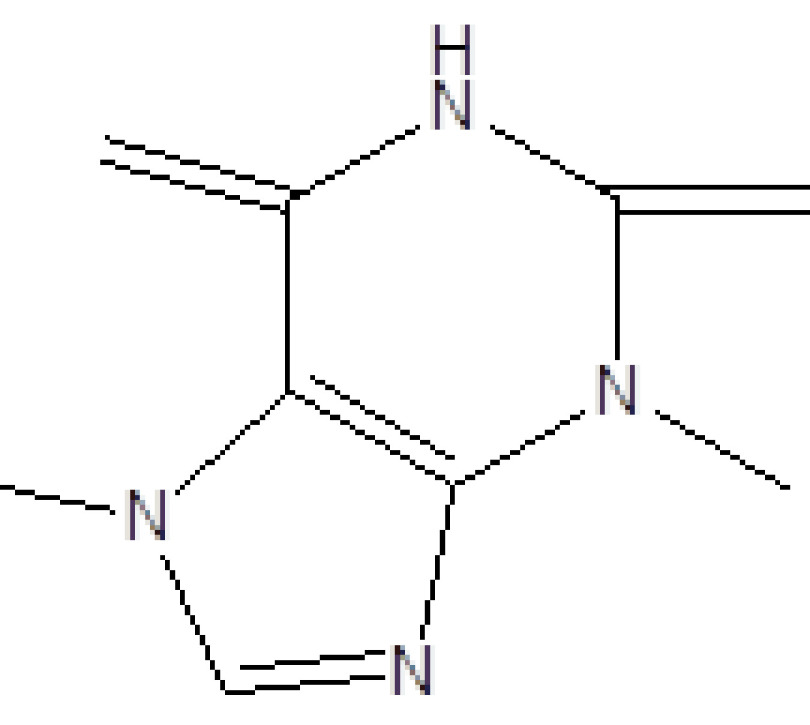
9	22.200	0.53	Methyl octadecanoate	C_19_H_38_O_2_	298	112-61-8	Ester	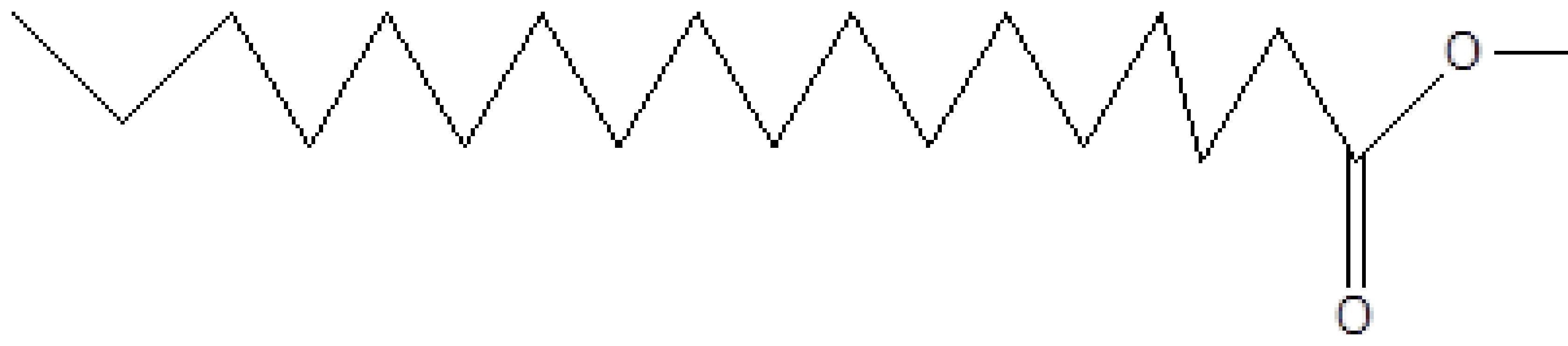
10	24.303	0.49	Hexadecadienoic acid, methyl ester	C_17_H_30_O_2_	266	56875-67-3	Ester	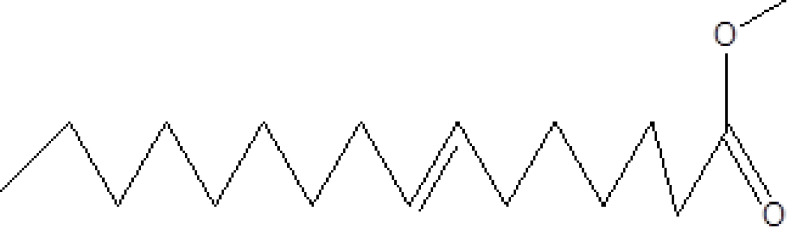

### 3.2. Antioxidant activity

**Figure 5:**
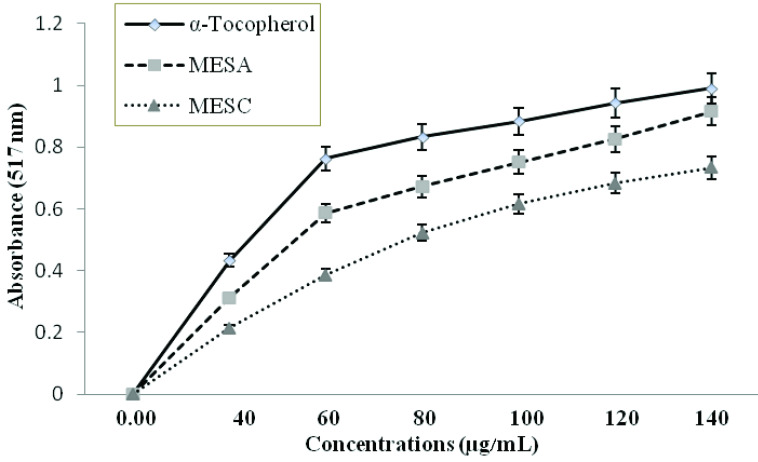
Dose-dependent scavenging of DPPH radicals by the methanolic extracts of *Smilax china* (MESC) and *Salix alba* (MESA) compared with standard drug α-tocopherol. Each value represents mean ±SD (n = 4). IC_50_ values of MESC, MESA, and α-tocopherol were 82 ±1.14, 57 ±1.75, and 42 ±1.18 μg/mL.

Antioxidant activities of MESC and MESA were assessed by the DPPH assay, in which the utilization of a stable free radical (DPPH) was measured. When a solution of DPPH (used as an indicator) is mixed with an antioxidant compound that donates a hydrogen atom, it contributes to the reduced form of diphenyl picrylhydrazine (nonradical). The color of the reaction mixture changed from purple to yellow and its absorbance was measured at a wavelength of 517 nm. The standard α-tocopherol compound was taken to compare the antioxidant activity of both extracts. The IC_50_ of DPPH scavenging activity was calculated graphically (Figure 5). Both MESC and MESA at a concentration of 100 μg/mL showed 63.5% and 78.0% activity, comparable to that of the positive control (α-tocopherol: 84.8%). It was found that the antioxidant activity of both extracts was directly proportional to the concentration of extracts. Lower IC_50_ values indicate higher antioxidant activity. Oxidative stress is the foremost aspect involved in neurogeneration. There is evidence that reactive oxygen (RO) and reactive nitrogen (NO) can be complicated in many neurodegenerative diseases like Parkinson’s disease, multiple sclerosis, Alzheimer’s disease, amyotrophic lateral sclerosis, Lewy body dementia, and vascular dementia. The antioxidant action may be attributed to the presence of phenolic compounds and terpenes. The phytoconstituents existing in the extracts verified by GC-MS are rich in antioxidant properties, which are responsible for neuroprotective activity [21].

## 4. Conclusions

The methanolic extracts of both *Smilax china* and *Salix alba* contain polar as well as nonpolar components, which were qualitatively identified by chemical tests and GC-MS analysis. The analysis exhibited varied bioactive compounds including alkaloids, terpenoids, pyrogallol, fatty acids, dienes, and different types of ester compounds. These components are reported to have certain pharmacological activities like antidiabetic, anthelminthic, antioxidant, antiepileptic, pesticidal, antigonorrheal, antipsoriatic, analgesic, antiinflammatory, and antirheumatic activity. GC-MS reports can also be used in the pharmaceutical industry to identify various biochemical markers in polyherbal extracts and the authentication/validation of individual plants. This study also confirmed that the anticipated antioxidant activity of methanolic extracts might be due to the presence of terpenoids, alkaloids, and unsaturated fatty acids. The elevated antioxidant activity of *Smilax china* (rhizome) and *Salix alba* (bark) extracts could be helpful in inhibiting or slowing the progress a variety of oxidative stress-related ailments.
